# Status and influencing factors of self-care among patients with chronic heart failure and their spouses from a dual perspective: a cross-sectional study

**DOI:** 10.3389/fcvm.2026.1818518

**Published:** 2026-10-08

**Authors:** Xiaoyan Wu, Yiyu Zhuang, Manjun Wang, Jinhua Jin, Jun Xie, Yuting Ke

**Affiliations:** Nursing Department, Sir Run Run Shaw Hospital, Zhejiang University School of Medicine, Hangzhou, Zhejiang, China

**Keywords:** chronic heart failure, dyad, mutuality, self-care, spouses

## Abstract

**Objective:**

To investigate the current status and influencing factors of self-care among chronic heart failure (CHF) patients and their spouses in China from a dyadic perspective, and to explore the interaction mechanisms between actor and partner variables.

**Methods:**

Using a cross-sectional design, 204 dyads of CHF patients and their spouses were recruited from a tertiary hospital in Zhejiang Province. Data were collected via an online questionnaire platform, with trained investigators assisting elderly participants unable to complete the survey on their own. Questionnaires used in this study included the general information questionnaire, Self-Care of Heart Failure Index, Health Literacy Scale, Mutuality Scale, and Social Support Rating Scale. Data were analyzed using multiple linear regression and the Actor-Partner Interdependence Model.

**Results:**

Median scores of all three self-care subscales for both patients and spouses were below the adequacy threshold of 70 points, with the Self-care Management dimension presenting the lowest subscale scores. Patient self-care scores were significantly higher than spouse contribution scores (*P* < 0.001). Multiple regression indicated that mutuality and CHF training experience were positively associated with self-care for the dyads. APIM analysis revealed that patient health literacy and spouse mutuality had significant actor-partner effects, influencing both their own and their partner's self-care.

**Conclusion:**

Self-care levels in CHF dyads were generally low. Clinical interventions should shift from an individualistic focus to a dyadic approach, specifically targeting the enhancement of patient health literacy and spouse mutuality to improve overall family-based disease management.

## 1 Introduction

Chronic heart failure (CHF) is the terminal stage of various cardiovascular diseases. It not only has high morbidity rates but also leads to high readmission and mortality rates due to the recurring nature of the illness (1). According to the latest data from the “Report on Cardiovascular Health and Diseases in China 2024”, the current number of cardiovascular disease cases in China has reached 330 million, with CHF patients accounting for approximately 8.9 million (2). The number of affected individuals had shown an explosive growth of about 50% over the past few years. This trend is particularly severe in the context of increasing population aging. Because heart failure patients remain in a state of living with the disease for a long time, frequent symptoms such as dyspnea, fatigue, and fluid retention not only seriously damage individual physiological functions but also bring heavy economic and psychological burdens to patients and their families (3). Beyond individual-level physiological consequences, CHF poses substantial public-health challenges accompanied by considerable societal and economic burdens. In globally ageing populations, informal spousal caregivers play a critical role of long-term disease management, contributing to reduced hospital readmissions and lower healthcare expenditure (4). Nevertheless, such informal care frequently imposes emotional and physical burdens on spousal caregivers (5). Accordingly, shifting heart failure management from a patient-centered clinical task toward family-oriented, socially supported care is essential to safeguard healthcare sustainability and social welfare.

The core goal of CHF secondary prevention is to reduce readmission and mortality rates through effective self-care (6). Although pharmacological and device-assisted treatments have made significant clinical progress, the long-term prognosis of CHF depends largely on the level of self-care within the family environment. CHF self-care is defined as a naturalistic decision-making process, encompassing daily behaviors to maintain physiological stability (e.g., taking medication as prescribed, restricting water and sodium intake, regular exercise) and effective recognition and response when symptoms worsen (e.g., increasing diuretics, seeking medical attention in time) (7). However, many empirical studies point out that the current status of self-care among CHF patients is generally not optimistic, with particularly poor performance in the dimensions of symptom recognition and response, directly leading to unplanned readmission (8).

For a long time, clinical nursing interventions have primarily focused on behavioral changes in individual patients. However, this research paradigm based on individualism ignores the social embedding of chronic disease management. In the context of Chinese family culture, the spouse is usually the patient's closest and longest-term caregiver, deeply involved in the patient's daily diet, medication supervision, and symptom observation (9). The level of caregiver support directly determines the continuity of patient self-care behavior; conversely, fluctuations in the patient's condition also affect the spouse's mental health and quality of life. This relational dimension determines whether a patient can adhere to secondary prevention plans in the long term (10).

In recent years, the academic community has begun to advocate for a “Dyadic Perspective” to re-examine chronic disease management, regarding the disease not merely as an individual's affliction but as a challenge confronted by the couple as a collective unit (9). The Theory of Dyadic Illness Management (TDIL) emerged in this context. Formally proposed by Lyons et al. in 2018, this theory emphasizes that the patient and their caregiver should be viewed as an interdependent dyad, influencing dyadic health outcomes through dyadic appraisal (agreement on disease cognition) and dyadic management behaviors (collaboration and interaction between the two parties) (11). TDIL focuses not only on individual-level variables but also emphasizes the core role of dyadic traits (such as mutuality between spouses) in the disease trajectory. The theory posits that protective or risk factors for disease management originate from individual, dyadic, and socio-cultural levels. A scoping review based on the Actor-Partner Interdependence Model (APIM) approach concluded that a healthy dyadic relationship and mutual self-care improved heart failure self-care and patient outcomes (12). Good relationships and healthy communication in heart failure dyads were crucial in managing heart failure of decreased mortality (13). Dyadic care interventions improved the behavioural, health outcomes of the heart failure patients and reduced caregiver burden (14). Therefore, it is necessary to explore the factors influencing self-care among patients with heart failure and their spouses.

Currently, although research on CHF self-care in China is on the rise, most of it is still confined to a single perspective and fails to fully uncover the interactive influence mechanisms of behavior and cognition between patients and spouses. The absence of this perspective may lead to ineffective nursing intervention strategies. Guided by the TDIL theory, this study adopted the APIM as a statistical analysis framework. The APIM can effectively address the non-independence issues in dyadic data. By distinguishing between actor effects (the individual's influence on themselves) and partner effects (the spouse's influence on the individual), it offers precise mathematical support for uncovering the interaction mechanisms between spouses regarding variables such as health literacy and mutuality. This study aimed to comprehensively analyze the current status of self-care among CHF patients and their spouses and identify key factors influencing the dyadic interaction process, thereby providing evidence for developing family-based, dyadic-collaborative nursing intervention programs.

## 2 Materials and methods

### 2.1 Study participants

This study included 204 couples of CHF patients and their spouses from a large tertiary general hospital in Zhejiang Province, China, using convenience sampling from February to May in 2024. The inclusion criteria were: Patients (1) married; (2) diagnosed with chronic heart failure (New York Heart Association classification II-IV); (3) communicated normally; (4) voluntarily signed the informed consent. Spouse (1) married; (2) caregiver of the patient; (3) communicated normally; (4) voluntarily signed the informed consent. The exclusion Criteria were: Patients (1) diagnosed with dementia or cognitive impairment; (2) diagnosed with acute heart failure; (3) diagnosed with serious diseases such as cerebrovascular and tumor. Spouse (1) diagnosed with dementia or cognitive impairment; (2) diagnosed with serious diseases such as cerebrovascular and tumor.

### 2.2 Study instruments

#### 2.2.1 General information questionnaire

A self-designed questionnaire was used to collect general information. Patients data on demographics included gender, age, employment, education level, medical insurance method, marriage duration, CHF self-care training experience, etc., and clinical characteristics included disease duration, number of hospitalizations last year, NYHA classification, other chronic diseases. Spouse data on demographics included gender, employment, education level, length of marriage, CHF self-care training experience, and whether they had chronic diseases.

#### 2.2.2 Self-care of heart failure index

The patient self-care was measured with Self-Care of Heart Failure Index, Version 6.2 (SCHFI 6.2) in three subscales: self-care maintenance, self-care management and self-care confidence (15). Each subscale was measured on a 4-point Likert scale and standardized to a 0−100 range, the subscale <70 was considered inadequate. A higher score indicated better self-care behaviors. The self-care of spouse caregivers was assessed with the Caregiver Contribution to Self-Care of Heart Failure Index, Version 1 (CC-SCHFI 1) (16). This instrument is identical to the SCHFI 6.2 in structure, content and scoring, with the only difference being that it is completed by caregivers.

#### 2.2.3 Health literacy scale

The patient health literacy was measured with Heart Failure Specific Health Literacy Scale (HFS-HLS) (17). It consisted of three subscales, namely functional health literacy, communicative health literacy, and critical health literacy, each item was measured on a 4—point Likert scale, a higher score indicated higher level of health literacy. The spouse health literacy was measured with Health Literacy Scale for Chronic Heart Failure Caregivers (18). It contained three subscales: health knowledge and awareness, health behavior, and health skills, each item was measured on a 5—point Likert scale, A score ≥105 was considered as indicating a high level of health literacy among caregivers. Notable differences exist between the two health literacy instruments in terms of dimensional structure and scoring. These two instruments were selected because they are the validated, disease-specific tools for their respective target populations in the Chinese context. However, they are not psychometrically equivalent, and direct cross-dyad comparison of raw scores should be approached with caution.

#### 2.2.4 Mutuality scale

The relationship between the patient and spouse was measured with Mutuality Scale (MS) (19). The scale comprised four dimensions: love and affection, reciprocity, sharing of pleasure, and values, each item was measured on a 5—point Likert scale. A score <2.5 was regarded as poor relationship.

#### 2.2.5 Social support rating scale

Social support was measured with Social Support Rating Scale (SSRS) (20). The scale consisted of three dimensions: objective support, subjective support and utilization of support. A higher score indicated better social support received by the individual.

### 2.3 Data collection and quality control

All surveys were administered by well-trained investigators via an online questionnaire platform. Investigators first explained the survey instructions in detail. For elderly participants with impaired eyesight, investigators read each questionnaire item aloud, recorded their answers into the online system on their behalf, and avoided any suggestive cues throughout the process. All participant responses were uniformly entered and stored in the online questionnaire system. We monitored the time spent on questionnaire completion and adopted double data entry for quality control. A total of 210 patient-spouse dyads were recruited, among which 204 valid paired questionnaires were retrieved, yielding an effective response rate of 97.1%.

### 2.4 Statistical analysis

Statistical analysis was performed using SPSS 26.0 and AMOS 23.0. Quantitative data following a normal distribution were described using mean ± standard deviation, while categorical data were described using number of cases and proportions. Quantitative variables not following a normal distribution were described using median and quantile interval. Comparisons between groups were performed using the Mann–Whitney *U* test or Kruskal–Wallis test, and paired comparisons were performed using paired samples t-test or signed-rank test. The Spearman method was used to analyze the correlation between quantitative variables. Multiple linear regression models were used to analyze the influencing factors of patient self-care ability and spouse's contribution to self-care. APIM was established to analyze the actor-partner effects of patient and spouse health literacy, mutuality, social support, and patient self-care and spouse contribution to self-care. Candidate predictors (including health literacy, mutuality, and social support) were initially evaluated using preliminary multiple linear regression analyses. To ensure model parsimony and avoid over-parameterization in structural equation modeling, only variables demonstrating statistically significant associations in multivariate regressions were retained as structural predictors in the final APIM. A *P* value < 0.05 was considered statistically significant.

### 2.5 Ethical considerations

Ethical approval for the study was granted by the Medical Ethics Committee of the affiliated hospital of authors' hospital (Approval Number: 20240432). All participants were provided with informed consent prior to their participation in the study. The data collected was anonymous and used solely for research purposes.

## 3 Results

### 3.1 Demographic and clinical profiles

Across the 204 patient-spouse dyads, the mean marriage duration was 37.3 ± 9.4 years, ranging from 10 to 57 years. Patients had a mean age of 61.4 years, 60.8% were male, 51.0% had primary education or lower, and 64.7% presented with NYHA classification III–IV heart failure. The median disease duration since confirmed CHF diagnosis was 8.5 (6.0, 12.0) months, with a range of 3−40 months. Spouses were predominantly female (60.8%), with a mean age of 60.78 years. Less than 10.0% of dyads had received CHF self-care training. The remaining demographic and clinical details are shown in Table 1.

**Table 1:** Baseline characteristic of CHF patients and their spouses (*n* = 204 pairs).

Variables	Groups	Patients, *n* (%)	Spouses, *n* (%)
Gender	Male	124 (60.8)	80 (39.2)
	Female	80 (39.2)	124 (60.8)
Level of education	Primary or lower	104 (51.0)	96 (47.1)
	Secondary/High school	73 (35.8)	82 (40.2)
	College or above	27 (13.2)	26 (12.7)
Employment	Employed	49 (24.0)	52 (25.5)
	Retired	63 (30.9)	76 (37.3)
	Unemployed	6 (2.9)	5 (2.4)
	Farmers	86 (42.2)	71 (34.8)
Medical insurance	New rural cooperative medical insurance	86 (42.1)	–
	Basic medical insurance for employees	105 (51.5)	–
	Basic medical insurance for urban and residents	13 (6.4)	–
Financial pressure	Light	83 (40.7)	86 (42.1)
	Medium	76 (37.2)	73 (35.8)
	Heavy	45 (22.1)	45 (22.1)
Times of hospitalization last year	0	56 (27.4)	–
	1	94 (46.1)	–
	2	31 (15.2)	–
	≥3	23 (11.3)	–
NYHA classification	II	72 (35.3)	–
	III	91 (44.6)	–
	IV	41 (20.1)	–
CHF self-care training	No	187 (91.7)	189 (92.7)
	Yes	17 (8.3)	15 (7.3)
Other chronic diseases	No	47 (23.0)	134 (65.7)
	Yes	157 (77.0)	70 (34.3)

NYHA, New York Heart Association; CHF, Chronic heart failure.

### 3.2 Self-care status of patient performance and spouse contribution

Patient self-care scores were significantly higher than spouse contribution scores (*P* < 0.001), especially in “Self-care Management”. Median values of every self-care subscale for the dyads were below the adequacy standard of 70 points (Table 2).

**Table 2:** Comparison of self-care scores between patients and their spouses [*M*(P25, P75)].

Dimension	Patients (*n* = 204)	Spouses (*n* = 204)	*Z*	*P*
Self-care maintenance	23.3 (13.3, 46.6)	23.3 (10.0, 40.0)	−7.532	<0.001
Self-care management	20.0 (0.0, 40.0)	15.0 (2.5, 30.0)	−4.017	<0.001
Self-care confidence	38.9 (19.4, 61.1)	36.1 (16.6, 61.1)	−3.639	<0.001
Total scores	78.6 (42.2, 145.0)	75.0 (35.0, 128.6)	−6.238	<0.001

### 3.3 Influencing factors

#### 3.3.1 Univariate analysis

##### 3.3.1.1 Univariate analysis of patient self-care

Statistically significant differences were found in self-care maintenance, self-care management, and self-care confidence among patients with different educational levels, occupational statuses, types of medical insurance, number of hospitalizations, and whether they had CHF self-care training experience (*P* < 0.05). Statistically significant differences were also found in self-care management among patients with different NYHA classifications (*P* < 0.05). Patients living with comorbid chronic conditions exhibited significantly higher self-care management scores compared to patients without additional chronic illnesses (*P* < 0.05). See Table 3 for details. Self-care maintenance, self-care management and self-care confidence were negatively correlated with age and marriage duration, and positively correlated with disease duration. See Table 4 for details.

**Table 3:** Results of univariate analysis of patient self-care [*M*(P25, P75)].

Variables	Groups	Self-care maintenance	Self-care management	Self-care confidence	Total scores
Gender	Male	26.7 (13.3, 45.0)	20.0 (0.0, 40.0)	38.9 (22.2, 61.2)	87.5 (45.6, 136.4)
	Female	21.7 (13.3, 50.0)	15.0 (0.0, 45.0)	33.4 (16.7, 61.2)	70.9 (35.3, 149.5)
*Z*		−0.079	−0.489	−0.753	−0.612
*P*		0.937	0.625	0.451	0.540
Education	Primary or lower	16.7 (10.0, 23.3)	10.0 (0.0, 22.5)	22.2 (11.1, 38.9)	48.9 (31.1, 77.0)
	Secondary/High school	36.7 (20.0, 50.0)	25.0 (5.0, 50.0)	55.6 (33.4, 61.2)	114.5 (65.6, 155.6)
	College or above	50.0 (40.0, 60.0)	45.0 (35.0, 55.0)	66.7 (55.6, 72.3)	163.9 (128.4, 182.8)
*H*		64.259	38.608	72.894	69.144
*P*		<0.001	<0.001	<0.001	<0.001
Employment	Employed	33.3 (23.3, 43.3)	20.0 (0.0, 40.0)	50.0 (33.4, 66.7)	113.4 (56.7, 137.8)
	Retired	46.7 (26.7, 56.7)	40.0 (15.0, 55.0)	55.6 (44.5, 72.3)	145.0 (86.2, 177.3)
	Unemployed	26.7 (13.3, 43.3)	12.5 (0.0, 25.0)	38.9 (38.9, 50.0)	86.4 (48.9, 113.9)
	Farmers	13.3 (10.0, 20.0)	10.0 (0.0, 20.0)	22.2 (11.1, 33.4)	45.0 (28.9, 69.5)
*H*		58.556	33.684	55.601	58.272
*P*		<0.001	<0.001	<0.001	<0.001
Medical insurance	New rural cooperative medical insurance	13.3 (10.0, 20.0)	10.0 (0.0, 20.0)	22.2 (11.1, 33.4)	44.5 (28.9, 66.7)
	Basic medical insurance for employees	43.3 (23.3, 53.3)	35.0 (10.0, 55.0)	55.6 (44.5, 72.3)	124.5 (79.5, 171.7)
	Basic medical insurance for urban and residents	30.0 (20.0, 46.7)	20.0 (0.0, 35.0)	27.8 (22.2, 50.0)	71.1 (45.6, 137.8)
*H*		63.204	31.358	67.755	65.959
*P*		<0.001	<0.001	<0.001	<0.001
Financial pressure	Light	30.0 (16.7, 50.0)	25.0 (5.0, 45.0)	50.0 (22.2, 61.2)	96.1 (47.8, 156.2)
	Medium	21.7 (13.3, 41.7)	15.0 (0.0, 35.0)	33.4 (16.7, 55.6)	69.2 (40.0, 123.1)
	Heavy	26.7 (13.3, 43.3)	20.0 (0.0, 35.0)	33.4 (22.2, 66.7)	79.5 (37.8, 135.0)
*H*		4.001	2.944	2.926	3.727
*P*		0.135	0.229	0.232	0.155
Times of hospitalization last year	0	20.0 (13.3, 41.7)	5.0 (0.0, 22.5)	27.8 (16.7, 55.6)	53.9 (35.6, 119.8)
	1	25.0 (13.3, 46.7)	15.0 (5.0, 40.0)	44.5 (16.7, 61.2)	78.4 (42.8, 137.3)
	2	26.7 (13.3, 50.0)	20.0 (0.0, 35.0)	38.9 (22.2, 55.6)	78.9 (44.5, 157.3)
	≥3	50.0 (23.3, 63.3)	55.0 (30.0, 70.0)	61.2 (50.0, 72.3)	171.7 (98.9, 197.3)
*H*		12.784	34.845	12.889	21.306
*P*		0.005	<0.001	0.005	<0.001
NYHA classification	II	20.0 (13.3, 40.0)	2.5 (0.0, 17.5)	30.6 (16.7, 55.6)	53.1 (35.3, 116.2)
	III	30.0 (13.3, 43.3)	25.0 (10.0, 45.0)	50.0 (22.2, 61.2)	98.9 (50.0, 145.0)
	IV	33.3 (13.3, 56.7)	25.0 (20.0, 60.0)	33.4 (16.7, 66.7)	90.6 (52.2, 179.5)
*H*		5.633	34.027	4.216	11.670
*P*		0.060	<0.001	0.121	0.003
Other chronic diseases	No	23.3 (13.3, 43.3)	5.0 (0.0, 30.0)	33.4 (22.2, 61.2)	56.7 (35.6, 135.0)
	Yes	26.7 (13.3, 50.0)	20.0 (5.0, 40.0)	38.9 (16.7, 61.2)	79.5 (45.6, 147.8)
*Z*		1.335	2.827	−0.160	1.384
*P*		0.182	0.005	0.873	0.166
CHF self-care training	No	23.3 (13.3, 43.3)	15.0 (0.0, 35.0)	33.4 (16.7, 55.6)	71.1 (39.5, 132.3)
	Yes	56.7 (50.0, 63.3)	55.0 (50.0, 60.0)	66.7 (55.6, 77.8)	179.5 (155.6, 205.6)
*Z*		5.074	5.045	3.957	4.922
*P*		<0.001	<0.001	<0.001	<0.001

NYHA, New York Heart Association; CHF, Chronic heart failure.

**Table 4:** Correlation analysis of patient self-care with age, marriage duration, and disease duration (*r_s_*).

Variables/Dimension	Self-care maintenance	Self-care management	Self-care confidence	Total scores
Age	−0.393**	−0.246**	−0.387**	−0.385**
Marriage duration	−0.350**	−0.177*	−0.379**	−0.345**
Disease duration	0.138*	0.321**	0.140*	0.203**

Disease duration refers to the time (in months) elapsed since the clinical confirmation of chronic heart failure.

* *P* < 0.05.

** *P* < 0.01.

##### 3.3.1.2 Univariate analysis of spouse contribution to self-care

Statistically significant differences were found in contribution to self-care maintenance, self-care management, and self-care confidence among spouses with different educational levels, occupational statuses, and whether they had CHF self-care training experience (*P* < 0.05). Spouses without chronic diseases had higher scores in contribution to self-care maintenance and self-care confidence than spouses with chronic diseases (*P* < 0.05). See Table 5 for details. Spouse contribution to self-care maintenance, self-care management and self-care confidence showed negative rank correlations with age and marriage duration. See Table 6 for details.

**Table 5:** Results of univariate analysis of spouse self-care contribution [*M*(P25, P75)].

Variables	Groups	Self-care maintenance	Self-care management	Self-care confidence	Total scores
Gender	Male	21.7 (6.7, 41.7)	12.5 (0.0, 30.0)	33.4 (16.7, 55.6)	75.9 (30.8, 123.9)
	Female	23.3 (13.3, 38.3)	15.0 (5.0, 35.0)	38.9 (16.7, 61.2)	75.0 (37.8, 130.9)
*Z*		1.074	0.510	0.440	0.850
*P*		0.283	0.610	0.660	0.395
Education	Primary or lower	10.0 (6.7, 16.7)	10.0 (0.0, 15.0)	16.7 (11.1, 36.1)	37.0 (25.6, 59.2)
	Secondary/High school	30.0 (20.0, 40.0)	20.0 (5.0, 35.0)	44.5 (27.8, 61.2)	92.0 (61.1, 137.8)
	College or above	48.3 (40.0, 53.3)	50.0 (30.0, 55.0)	72.3 (55.6, 72.3)	158.1 (136.2, 182.3)
*H*		84.333	53.157	76.885	83.469
*P*		<0.001	<0.001	<0.001	<0.001
Employment	Employed	33.3 (20.0, 40.0)	22.5 (2.5, 37.5)	44.5 (27.8, 63.9)	95.0 (57.2, 138.4)
	Retired	33.3 (20.0, 46.7)	25.0 (10.0, 42.5)	50.0 (27.8, 61.2)	108.4 (60.9, 143.4)
	Unemployed	13.3 (6.7, 36.7)	5.0 (0.0, 30.0)	50.0 (38.9, 61.2)	58.4 (52.3, 138.9)
	Farmers	10.0 (6.7, 16.7)	10.0 (0.0, 15.0)	16.7 (11.1, 27.8)	34.5 (23.3, 46.1)
*H*		65.352	30.698	51.064	58.955
*P*		<0.001	<0.001	<0.001	<0.001
Financial pressure	Light	25.0 (10.0, 43.3)	15.0 (5.0, 35.0)	44.5 (16.7, 61.2)	85.0 (36.1, 132.3)
	Medium	20.0 (10.0, 33.3)	15.0 (0.0, 30.0)	27.8 (16.7, 55.6)	58.4 (34.5, 113.9)
	Heavy	23.3 (10.0, 36.7)	15.0 (5.0, 30.0)	33.4 (16.7, 61.2)	72.8 (37.2, 133.4)
*H*		1.781	0.840	3.303	2.086
*P*		0.410	0.657	0.192	0.352
Other chronic diseases	No	26.7 (10.0, 40.0)	20.0 (0.0, 35.0)	44.5 (16.7, 61.2)	86.2 (37.8, 136.1)
	Yes	16.7 (6.7, 33.3)	10.0 (5.0, 30.0)	25.0 (11.1, 50.0)	50.3 (31.1, 117.3)
*Z*		−2.135	−1.025	−2.705	−2.191
*P*		0.033	0.306	0.007	0.028
CHF self-care training	No	20.0 (10.0, 36.7)	15.0 (0.0, 30.0)	33.4 (16.7, 55.6)	61.1 (34.5, 118.4)
	Yes	50.0 (33.3, 60.0)	50.0 (40.0, 55.0)	72.3 (44.5, 72.3)	172.3 (122.8, 188.9)
*Z*		4.939	5.196	3.840	4.880
*P*		<0.001	<0.001	<0.001	<0.001

**Table 6:** Correlation analysis of spouse self-care contribution with age, marriage duration (*r_s_*).

Variables/Dimension	Self-care maintenance	Self-care management	Self-care confidence	Total scores
Age	−0.423**	−0.335**	−0.350**	−0.408**
Marriage duration	−0.422**	−0.294**	−0.380**	−0.409**

** *P* < 0.01.

#### 3.3.2 Multiple linear regression

##### 3.3.2.1 Multiple linear regression of patient self-care

Multivariate regression analysis indicated that patient health literacy (*β* = 0.355, *P* < 0.001), patient mutuality (*β* = 0.353, *P* < 0.001), spouse mutuality (*β* = 0.197, *P* < 0.05), NYHA classification (*β* = 0.143, *P* < 0.001), and CHF self-care training experience (*β* = 0.079, *P* < 0.05) were positively associated with patient self-care, as shown in Tables 7, 8.

**Table 7:** Assignment of independent variables.

Variables	Assignment
Education	1 = Primary or lower; 2 = Secondary/High school; 3 = College or above
Times of hospitalization last year	1 = 0; 2 = 1; 3 = 2; 4 = 3 or above
NYHA classification	1 = NYHA II; 2 = NYHA III; 3 = NYHA IV
CHF training experience	0 = No; 1= Yes
Employment	Using “farmer” as a reference, dummy variables are set up: employed = (1, 0, 0); retired = (0, 1, 0); unemployed = (0, 0, 1).
Medical insurance	Using “new rural cooperative medical care” as a reference, dummy variables are set up: basic medical insurance for mployees = (1, 0); basic medical insurance for urban and rural residents = (0, 1).
Age	Input of original value variable
Length of marriage	Input of original value variable
Disease duration	Input of original value variable
Patient health literacy	Input of original value variable
Patient mutuality	Input of original value variable
Patient social support	Input of original value variable
Spouse health literacy	Input of original value variable
Spouse mutuality	Input of original value variable
Spouse social support	Input of original value variable

**Table 8:** Results of multiple linear regression for patient self-care (*n* = 204).

Variables	*B*	*SE*	*β*	*t*	*P*	VIF
Constant	−92.561	9.716	–	−9.526	<0.001	–
Patient mutuality	26.088	6.248	0.353	4.176	<0.001	6.324
Patient health literacy	2.415	0.334	0.355	7.236	<0.001	2.123
Spouse mutuality	14.245	5.562	0.197	2.561	0.011	5.223
New York Heart Association classification	11.901	2.845	0.143	4.184	<0.001	1.030
CHF training experience (Yes)	17.451	8.057	0.079	2.166	0.032	1.189

##### 3.3.2.2 Multiple linear regression of spouse contribution to self-care

Multivariate regression analysis indicated that spouse mutuality (*β =* 0.268, *P* < 0.05), spouse health literacy (*β =* 0.237, *P* < 0.05), spouse education (*β =* 0.21, *P* < 0.001), patient mutuality (*β =* 0.201, *P* < 0.05) and CHF self-care training experience (*β =* 0.130, *P* < 0.05) were significant predictors of spouse contribution to self-care, as shown in Tables 7, 9.

**Table 9:** Results of multiple linear regression for spouse contribution to self-care (*n* = 204).

Variables	*B*	*SE*	*β*	*t*	*P*	*VIF*
Constant	−101.229	13.434	–	−7.535	0.000	–
Spouse health literacy	0.661	0.208	0.237	3.179	0.002	3.960
Spouse mutuality	17.517	5.729	0.268	3.057	0.003	5.491
Patient mutuality	13.360	6.218	0.201	2.149	0.033	6.206
Education	16.833	4.638	0.213	3.629	0.000	2.454
CHF training experience (Yes)	27.275	8.448	0.130	3.228	0.001	1.155

Given the high inter-correlation between patient and spouse reported mutuality (*r* = 0.90, VIF = 6.324), two potential explanations are noted. First, this association stems from the dyadic nature of our sample. Mutuality between patient and spouse is theoretically intertwined within marital relationships, yielding their strong empirical correlation and elevated VIF values. Second, such dyadic interdependence is explicitly modelled via the covariance path between dyad-level predictors within the APIM framework. Since all VIF values were below 10.0 and path-parameter standard errors remained small (SE = 0.048−0.067), multicollinearity did not distort parameter estimates or inflate standard errors.

### 3.4 APIM actor-partner effects

In preliminary multiple linear regression analyses, social support exhibited no statistically significant associations with self-care related outcomes. Accordingly, social support was excluded from the subsequent structural path analysis, health literacy and mutuality were retained as core predictors in the final APIM. The final model achieved satisfactory fit: *χ*^2^/*df* = 1.066, RMSEA = 0.018. Significant cross-dyad actor-partner effects are presented in Table 10 and Figure 1.

**Table 10:** APIM path results: standardized coefficients for actor and partner effects.

Path (Independent variable → Dependent variable)	Effect type	*β*	*SE*	*P*
Patient literacy → Patient self-care	Actor	0.423	0.048	<0.001
Patient mutuality → Patient self-care	Actor	0.250	0.061	<0.001
Spouse mutuality → Patient self-care	Partner	0.282	0.065	<0.001
Patient literacy → Spouse contribution	Partner	0.252	0.067	<0.001
Spouse literacy → Spouse contribution	Actor	0.300	0.052	<0.001
Spouse mutuality → Spouse contribution	Actor	0.374	0.054	<0.001

**Figure 1:**
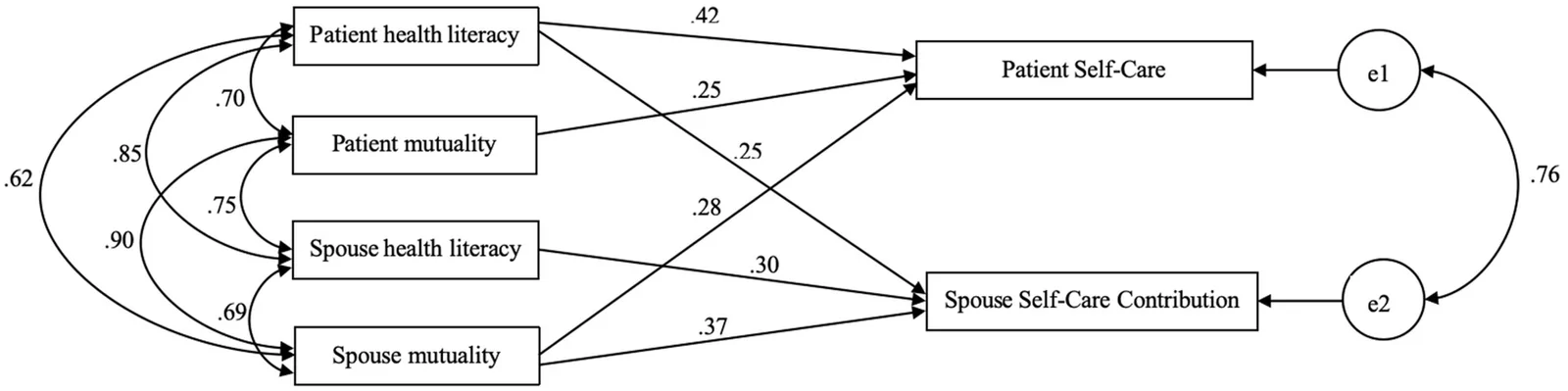
Actor-Partner Interdependence Model of patient self-care and spouse self-care contribution. Standardized path coefficients are presented for all significant paths. e1 and e2 denote residual error terms for patient self-care and spouse self-care contribution, respectively. The six pairwise inter-correlations among predictors shown in the figure are as follows: patient health literacy ↔ patient mutuality = 0.70; patient health literacy ↔ spouse health literacy = 0.85; patient health literacy ↔ spouse mutuality = 0.62; patient mutuality ↔ spouse health literacy = 0.75; patient mutuality ↔ spouse mutuality = 0.90; spouse health literacy ↔ spouse mutuality = 0.69.

## 4 Discussion

### 4.1 Current status of CHF self-care from a dual perspective

In this study, median scores of all three self-care subscales for both patients and spouses were below the adequacy threshold of 70 points, indicating both the CHF patients and their spouses possess a low level of self-care capability, which aligns with the results of previous study (21). Of the three dimensions, both patients and spouses scored lowest in “self-care management”. This dimension includes items such as the time required to recognize a heart failure episode and the measures taken during an episode. Low scores in this dimension indicated that patient and their spouses had weak capabilities in monitoring and managing CHF symptoms, lacking theoretical knowledge and skills of self-care. This study showed that patient scores were higher than spouses (*P* < 0.001). It might be primarily related to the fact that CHF patients have severe conditions and experience repeated hospitalizations, allowing patients to accumulate more self-care experience (22). This suggests nurses should adopt a dual perspective, viewing patients and their spouses as a whole, and increase standardized CHF self-care training for both, enabling them to promptly detect heart failure symptoms and quickly implement appropriate measures to control the condition.

### 4.2 Influencing factors

This study showed that for every one level increase in the NYHA classification, the patient self-care score increased by 11.901 points, indicating that the worse the heart function, the better patient self-care ability. A high NYHA classification indicates that the patient's condition is serious and requires frequent hospitalization to control the condition and relieve symptoms. These medical experiences help patients better understand their condition and the key points of heart failure care, thereby improving the patient self-care ability (23). The spouse's education level was found to positively affect spouse contribution to self-care. Generally, individuals with higher education levels tend to have more economic resources to cope with financial pressure caused by the disease (24). At the same time, individuals with higher education levels possess relatively stronger learning capabilities, which is beneficial for guiding patients in self-care. Possessing the experience of CHF self-care training was also a significant factor. Through various forms of health education, patients and their spouses acquire knowledge and skills for CHF self-care. Studies (25, 26) confirmed that the main measure to improve the self-care ability of patients was to conduct effective health education and training.

This study showed that health literacy was positively affect self-care of both patient and their spouse. Health literacy refers to the ability of an individual to obtain and understand health information and to use this information to maintain and promote their own health (27). Health literacy is an important contributing factor to self-care in patients with heart failure (28). People with high health literacy have stronger learning ability, communication ability and initiative, and therefore can acquire more knowledge and skills, while also being aware of the importance of disease self-care (29). The study also found that the higher quality of mutuality between patient and spouse, the better self-care ability they possess. The mutuality refers to the mutual relationship between the couple, which reflects the positive qualities of their relationship, with positive emotions (19). Couples have the higher level of mutuality, the less negative emotions they experience during the illness and treatment process, which helps the patient and their spouse to communicate well, therefore, the spouse contribution to the patient's care is also higher (30). This suggests that, in addition to providing health education on CHF, nurses also need to assess the quality of mutuality between the couples to provide targeted support.

### 4.3 APIM actor-partner effects

This study showed that patient health literacy positively influenced their own self-care and their spouse self-care contribution, it was an actor-partner effect. Meanwhile, spouse health literacy positively influenced their own self-care contribution, producing only a partner effect. Therefore, the positive interactive effect of patient health literacy on the couples' self-care abilities is more significant. Self-care in heart failure is essentially a series of complex, immediate decision-making processes (7). As the first perceiver of physical symptoms, patient health literacy directly determines their sensitivity and ability to interpret early warning signs, such as peripheral edema and shortness of breath. Furthermore, symptom perception is private, and spousal intervention may be delayed. When patients possess sufficient health literacy, they can translate physiological changes into therapeutic decisions, such as adjusting the diuretic dosage or seeking timely medical attention (28). The study (31) indicated that the patient's own cognitive level was the cornerstone of intrinsic motivation. High health literacy enables patients to understand the logical connection between disease and treatment option, while spousal health literacy primarily serves as external supervision. In the long-term management of chronic diseases, behavioral changes based on external supervision lack resilience, while behavioral internalization based on self-cognition is more enduring. In this study, similar to patient health literacy, spouse mutuality also exhibited an actor-partner effect, which means that the positive interactive effect of spouse mutuality on couples' self-care abilities is more significant. A high level of spouse mutuality is a driving force for overcoming caregiver burden (32). When spouses feel a strong emotional connection with patients, they are more willing to view the patients' illness as a shared family problem and proactively invest energy in self-care contributions, such as symptom monitoring and medication management. A harmonious relationship between couples creates a low-stress, high-support environment for patients' behavioral change. APIM model analysis suggested that future chronic disease management strategies should focus more on interventions that enhance patient health literacy and promote spouse mutuality.

### 4.4 Implications

This study provides practical insights for healthcare providers in Chinese hospitals. First, for clinical practice, nurses should transition from patient-centered support to dyadic-centered interventions. Training programs should be standardized and involve both the patient and the spouse as a collective unit. Second, regarding of training and education, increasing the accessibility of CHF self-care training is critical, as currently less than 10% of dyads have received formal education. Specific emphasis must be placed on “Self-care Management” skills, such as timely symptom recognition and emergency response (e.g., diuretic adjustment), where dyads currently perform most poorly. Third, from a dual perspective, since spouse mutuality significantly impacts both parties of couple, nursing strategies should include relationship-building exercises and emotional support to improve self-care capability.

### 4.5 Limitations

This study has several limitations. Single-site convenience sampling limits generalizability. Distinct health-literacy scales for patients and spouses require cautious result interpretation. Online self-reports may introduce recall bias among older adults. Though mutuality of patient and spouse showed no severe multicollinearity, their close dyadic association may limit power to separate independent actor-partner effects, corresponding path coefficients should therefore be interpreted with caution. Being cross-sectional, it cannot infer causality. Future multi-center randomized sampling across varied regions and longitudinal tracking designs are warranted to validate causal pathways.

## 5 Conclusion

This study demonstrates insufficient self-care capacity in both patients with chronic heart failure and their spouses, with symptom management representing the weakest dimension. Mutuality and CHF training experience were positively associated with self-care for the dyads. The results highlight the interdependent characteristics of patient-spouse chronic disease management. Patient health literacy and spouse mutuality are core influencing factors that simultaneously generate actor and partner effects on dyadic self-care capability. To optimize long-term clinical prognosis and lower hospital readmission risks, clinical nursing practice should shift from patient-only interventions to couple-centered collaborative programs. Targeted strategies focused on improving patient health literacy and spouse mutuality are recommended to advance family-based heart failure disease management.

## Data Availability

The original contributions presented in the study are included in the article/Supplementary Material, further inquiries can be directed to the corresponding author/s.
